# Study on Frost Resistance of Recycled Rubber Straw Concrete Using Particle Swarm Optimization Enhanced Artificial Neural Networks

**DOI:** 10.3390/polym16223191

**Published:** 2024-11-17

**Authors:** Qijing Xia, Yongcheng Ji

**Affiliations:** College of Civil Engineering and Transportation, Northeast Forestry University, Harbin 150040, China; qijingxia2000@163.com

**Keywords:** rubber concrete, straw powder, mechanical prediction, particle swarm optimization (PSO) algorithm, genetic algorithm (GA), BP neural network model

## Abstract

Rubber particles and straw powder were used to prepare recycled rubber straw concrete, and the freeze–thaw test was conducted on the recycled rubber straw concrete using the quick-freezing method. The frost resistance of the recycled rubber straw concrete was evaluated by determining the relative dynamic modulus of elasticity, the rate of mass loss, and the flexural strength of the recycled rubber straw concrete in the process of freezing and thawing. SEM was used to observe the microstructure of the recycled rubber straw concrete after the freezing and thawing process. SEM observed the microstructure of recycled rubber straw concrete after freezing and thawing. The effect and mechanism of rubber admixture and straw admixture on the frost resistance of concrete were investigated by microanalysis. Based on the experimental data, the particle swarm algorithm and genetic algorithm were used to optimize the BP neural network to establish the prediction model of recycled rubber straw powder, and the results show that the PSO-BP neural network prediction model established in this paper has good accuracy and stability. It has a good prediction effect on the flexural strength and the number of freeze–thaw cycles of recycled rubber straw concrete under different mixing ratios.

## 1. Introduction

With the adjustment of China’s industrial structure and the improvement of the population’s living conditions, a large surplus of straw production has emerged. Although the utilization efficiency of straw shows a steady upward trend, the proportion of straw burning is still considerable, and this phenomenon has significantly become one of the primary triggers of atmospheric pollution globally [1]. Every year, there are nearly one billion waste tires worldwide, of which only 50% are recycled, and the rest end up in landfills, thus threatening the environmental system. With the environmental protection concept and resource utilization technology vigorously improved, the application field of corn stover and recycled rubber is gradually expanding [2,3,4,5]. Therefore, using straw and rubber in concrete is an effective way to reduce the mass dumping of both and, at the same time, mitigate the harmful effects of waste dumping.

Numerous scholars have thoroughly studied the effects of straw and rubber admixtures on the frost resistance of concrete, especially straw concrete and rubber concrete, which have been extensively studied [6,7,8,9,10]. Yang et al. [11] investigated the frost resistance of straw fiber concrete with different lengths and volumetric dosages under freeze–thaw cycle damage. The study showed that straw fibers can enhance the frost resistance of concrete. Feng et al. [12] incorporated alkali-treated straw fibers into concrete, and the study showed that an appropriate amount of alkali-treated straw fibers could reduce the internal porosity of concrete and enhance the frost resistance of concrete. According to the study, incorporating rubber into cementitious mixtures can improve the resistance of cementitious composites to freeze–thaw action. Chen et al. [13] showed that the incorporation of rape straw could reduce the thermal conductivity of concrete and improve the thermal insulation performance of concrete. The improvement effect of powdered straw is better than that of striped straw. Yan Yan [14] et al. showed that high-admixture rubberized concrete has better frost resistance than ordinary concrete and the advantage of frost resistance of rubberized concrete increases with the increase in the number of freeze–thaw cycles. Pham et al. [15] showed that rubber’s high energy absorption and hydrophobicity make the frost resistance of concrete better than that of ordinary concrete under freeze–thaw conditions.

Compared with ordinary cement concrete, recycled rubber straw concrete is mixed with straw powder and rubber particles, and the various properties of concrete have changed to different degrees, making frost durability more complex. It is more challenging to establish theoretical models. The BP neural network has been widely used in mortar, concrete, and other concrete strength prediction because of its advantages of nonlinear mapping ability, flexible network structure, and high prediction accuracy. The network is used for the prediction of concrete strength, such as mortar and concrete, but its prediction accuracy is affected by factors such as input layer, initial weights, and thresholds, which leads to a decrease in prediction accuracy. Some scholars have improved the BP neural network by using PSO and GA algorithms and enhanced the prediction ability of the BP model through the global search ability of the two algorithms, which has achieved good prediction results [16,17,18,19,20,21]. GA and PSO were selected due to their proven effectiveness in handling complex optimization problems related to material science [22]. However, there are fewer studies on recycled rubber straw concrete, and the research on the anti-freezing performance of recycled rubber straw concrete and the life prediction model needs to be deepened. Therefore, this paper optimizes the BP neural network by introducing PSO and GA algorithms and compares the prediction ability of the PSO-BP optimization model, GA-BP optimization model, and BP model on the antifreeze performance of recycled rubber straw concrete. The results show that the correlation coefficients of the PSO-BP optimization model, GA-BP optimization model, and BP model test set are 0.97, 0.94, and 0.93, respectively, which further enrich the prediction research on the antifreeze performance of recycled rubber straw concrete. At the same time, this study conducted the microstructural analysis on the recycled rubber straw concrete using the SEM test, which helps to understand the effect of the silane coupling agent on the frost resistance and life span prediction model of recycled rubber straw concrete. The microstructural analysis of recycled rubber straw concrete by SEM test in this paper can help to recognize the micro-mechanism of the effect of silane coupling agent on the performance of recycled rubber straw concrete.

## 2. Materials and Methods

### 2.1. Materials

**Figure 1:**
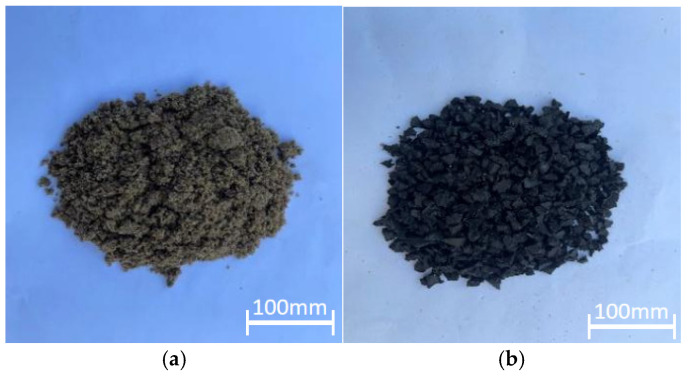
Experimental materials: (**a**) corn straw powder; (**b**) rubber particle.

Cement using P.O. 42.5 ordinary silicate cement; coarse aggregate selection of crushed stone, according to the grading requirements of the particle size, can be divided into two kinds: 5~10 mm, 10~20 mm, the mixing ratio of 3:7; fine aggregate selection of natural river sand, for the sand, fineness modulus 2.3; silane coupling agent selection of KH570, Dongguan Kangjin New Material Technology Co. (Dongguan, China) Straw selection of Heilongjiang local corn stalks by mechanical chopping fine grinding processed from 20 mesh corn stalks as shown in Figure 1a; Recycled rubber particles selection of Sichuan Huayi Rubber Company Limited (Chengdu, China) processing of 3–6 mm waste tire rubber particles, as shown in Figure 1b.

### 2.2. Experimental Design

This experimental design uses rubber admixture and corn stover powder admixture as the main research parameters. The plain concrete (PC) specimens were designed according to the strength class C30, with a water–cement ratio of 0.4; rubber particles were added by equal volume replacement of fine aggregate, with substitution rates of 0%, 10%, 20%, and 30%, respectively.

Corn stover was externally admixed with 0%, 2%, 4%, and 6% mass fractions; a total of 16 sets of specimens were designed. Where PC stands for normal concrete, RC stands for rubber concrete, MRC stands for modified rubber concrete, MRSPC stands for modified rubber straw powder concrete, and the number after it represents the numerical part of the corresponding admixture percentage, e.g., MRC-10 stands for modified rubber particles admixture of 10%, MRSPC20-2 stands for modified rubber particles admixture of 20%, and straw powder admixture of 2%. The mix proportions are shown in Table 1.

The instrument used for the freeze–thaw cycle test is the freeze–thaw cycle machine. The freeze–thaw cycle test process refers to the “Test Method for Long-Term Performance and Durability of Ordinary Concrete” (GB/T50082-2019 [23]) for rapid freeze–thaw test, and the number of freeze–thaw cycles is set to 0, 25, 50, 75 and 100 times. The specimens for freeze–thaw cycles were immersed in water at (20 ± 2) °C for four days in advance, and their mass and transverse fundamental frequency were measured in the water-saturated state. After the start of the test, every 25 freeze–thaw cycles, check the appearance, weighing, and measurement of the transverse fundamental frequency until the corresponding number of cycles stops. To complete a freeze–thaw cycle takes 4 h, with a freeze–thaw cycle temperature range of −15~10 °C, warming and cooling each need 1 h, and freezing and thawing time lasts about one hour each. The specimens that had experienced the freeze–thaw cycle test were carried out using the Test Method for Strength of Cementitious Sand (ISO Method) (GB/T 17671-2020 [24]). The flexural strength was measured. The loading rate for flexural strength was (50 ± 10)N-s-1, and that for compressive strength was (2400 ± 200)N-s-1.

## 3. Results and Discussion

### 3.1. Apparent Deterioration of Recycled Rubber Straw Concrete

**Figure 2:**
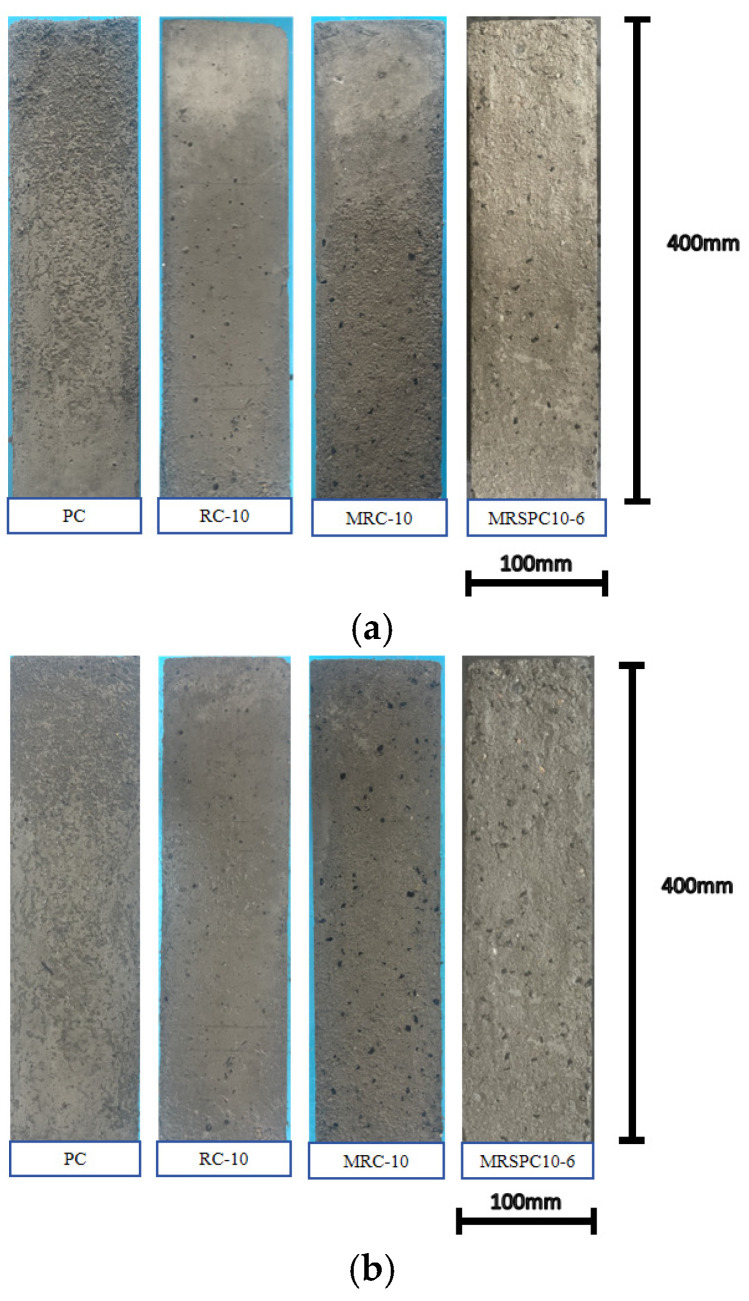

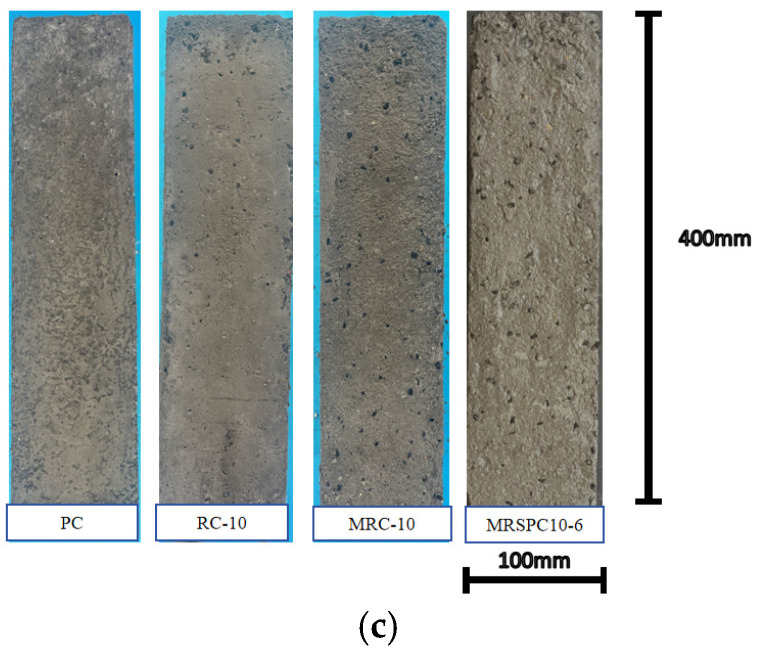
Recycled rubber straw concrete surface morphology: (**a**) 25 freeze–thaw cycles; (**b**) 50 freeze–thaw cycles; and (**c**) 100 freeze–thaw cycles.

Figure 2 shows the surface changes in recycled rubber straw concrete during freeze–thaw cycles. From the figure, it can be seen that at 25 times of freeze–thaw cycle, all the specimens showed the phenomenon of surface mortar spalling, and with the increase in the number of freeze–thaw cycles, the phenomenon of surface mortar spalling was intensified; after 50 times of freeze–thaw cycle, the surface of the specimens showed a large surface area of exposed coarse aggregate. For the modified rubber concrete specimens, under different freeze–thaw cycles, the overall surface condition was significantly better than that of rubber concrete, and the surface morphology of recycled rubber straw concrete mixed with straw powder was better than that of rubber concrete. Incorporating straw powder can enhance the spalling resistance of concrete and improve its frost resistance.

### 3.2. Mass Loss Rate of Recycled Rubber Straw Concrete

**Figure 3:**
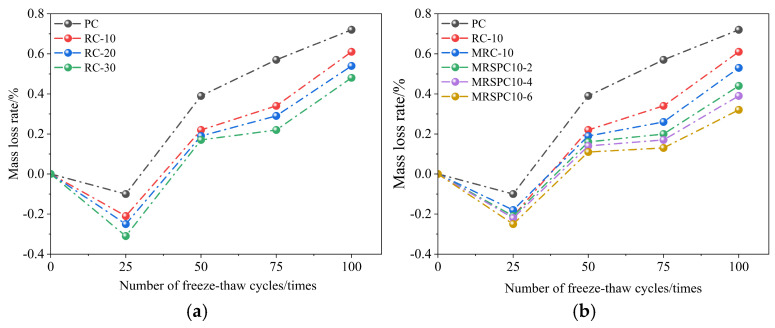
Mass loss rate of recycled rubber straw concrete after different freeze–thaw cycles: (**a**) different rubber doping and (**b**) different straw doping.

The mass loss rate of recycled rubber straw concrete under the action of freeze–thaw cycles versus the number of freeze–thaw cycles is shown in Figure 3. As shown in Figure 3a, some micro-cracks or pores on the surface of concrete specimens do not show an apparent slurry denudation phenomenon at the early stage of the freeze–thaw cycle. The water in the surrounding environment enters these initial cracks or pores inside the specimens, resulting in a slight increase in the quality of the specimens. After 25 freeze–thaw cycles, the damage inside the concrete gradually accumulated. Some micro-cracks or pores on the surface of concrete specimens showed slurry denudation. The slurry denudation phenomenon became gradually significant with the increase in freeze–thaw cycles. The mass loss rate of ordinary concrete was significantly higher than that of RC specimens with increased freeze–thaw cycles. The mass loss rate of RC-10 with 10% rubber dosage was significantly higher than that of RC-20 with 20% rubber dosage, which indicated that, on the one hand, rubber particles could absorb and disperse the stress generated in the freeze–thaw process and reduce the micro-cracks in the concrete. On the other hand, the deformation ability of rubber particles makes the cement easier to adapt to the deformation caused by the freeze–thaw cycle, thereby reducing the loss of surface cement slurry. As can be seen from Figure 3b, the mass loss rate of modified MRC-10 is significantly higher than that of unmodified RC-10, indicating that the freeze–thaw resistance of modified recycled rubber concrete mixed with modified silane coupling agent KH570 is better than that of unmodified recycled rubber concrete. With the increase in straw admixture, the mass loss rate of modified recycled rubber straw concrete was significantly reduced due to the increase in freeze–thaw number. Under the action of the freeze–thaw cycle, both the surface and the interior of the concrete will produce cracks; the straw absorbs the water infiltrated into the cracks of the concrete, which leads to the increase in the quality of the straw-mixed recycled rubber straw concrete, the quality of the mass loss rate was reduced.

### 3.3. Relative Dynamic Modulus of Elasticity of Recycled Rubber Straw Concrete

**Figure 4:**
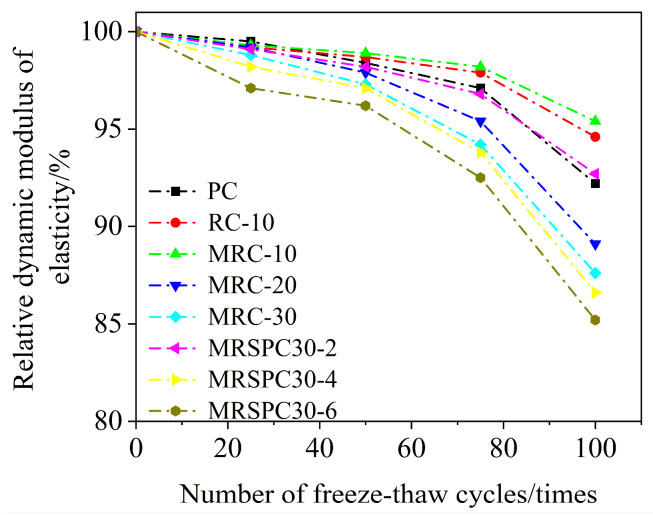
Relative dynamic modulus of elasticity of recycled rubber straw concrete after different freeze–thaw cycles.

Under different numbers of freeze–thaw cycles, the relative dynamic elastic modulus of some of the recycled rubber straw concrete with the number of freeze–thaw cycles is plotted according to the test data, as shown in Figure 4.

Figure 4 shows recycled rubber concrete’s relative kinetic elastic modulus, which shows an overall decreasing trend with the increase in freeze–thaw cycles. Under the same number of freeze–thaw cycles, recycled rubber concrete’s relative kinetic elastic modulus decreases more rapidly with the increase in rubber particle doping. At 50 times freeze–thaw cycles, the relative dynamic elastic modulus of RC-10 and PC was 98.7% and 98.4%, respectively. The relative dynamic elastic modulus of RC-10 was greater than that of PC at 75 times freeze–thaw cycles and 100 times freeze–thaw cycles, which indicated that the appropriate amount of recycled rubber particles slowed down the decline of the relative dynamic elastic modulus of concrete. The relative dynamic elastic modulus of the modified recycled rubber concrete was higher than that of the recycled rubber concrete during 25 to 100 cycles of freeze–thaw cycles, indicating that the modified recycled rubber concrete mixed with recycled rubber particles modified by silane coupling agent KH570 has better frost resistance. At 100 freeze–thaw cycles, the relative dynamic elastic modulus of MRC-30, MRSPC30-2, MRSPC30-4, and MRSPC30-6 was 87.6%, 92.7%, 87.5%, and 85.2%, respectively, the results show that when the content of straw is 2%, the relative dynamic elastic modulus of recycled rubber straw concrete is the highest. With the increase in the content of straw, the relative dynamic elastic modulus of recycled rubber straw concrete gradually decreases. The relative dynamic elastic modulus of MRSPC30-4 and MRSPC30-6 is lower than that of MRC when the straw content is 4% and 6%. It can be seen that the frost resistance of 2% recycled rubber straw concrete is the best, while the frost resistance of 4% and 6% recycled rubber straw concrete is adversely affected.

### 3.4. Flexural Strength of Recycled Rubber Straw Concrete

**Figure 5:**
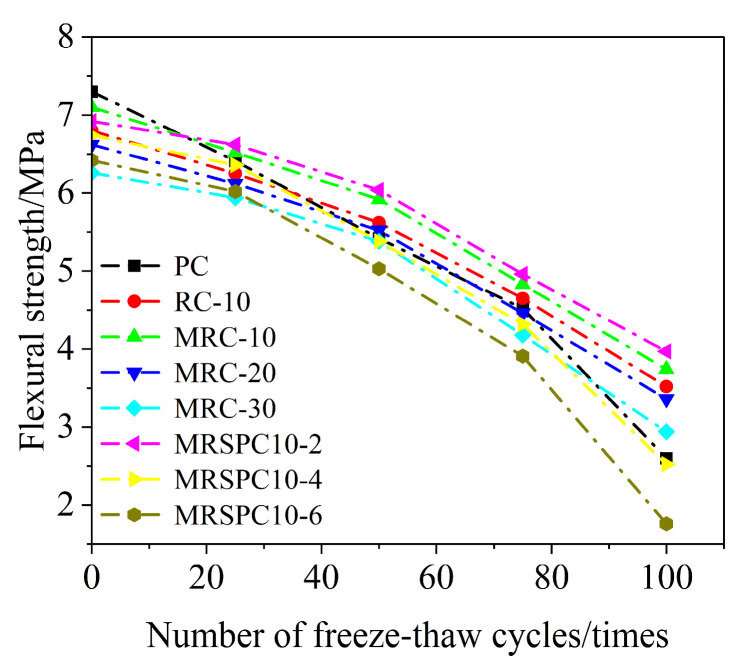
Flexural strength of recycled rubber straw concrete after different freeze–thaw cycles.

After every 25 freezing and thawing cycles, the specimens were tested for flexural strength, and the change curves of flexural strength under different numbers of freezing and thawing cycles are shown in Figure 5.

Figure 5 shows that the flexural strength of rubber concrete is lower than that of ordinary concrete (PC) at 0 times of freeze–thaw cycles, which is due to the hydrophobicity of rubber particles that makes the hydration of cement particles around the rubber particles insufficient, resulting in the weak transition zone of the interface between the rubber and the cementitious, and the flexural strength is reduced. The improvement of the flexural strength of concrete by rubber and straw is more evident after 50 times of freeze–thaw cycles. With the increase in freeze–thaw cycles, the flexural strength of rubberized concrete is gradually higher than that of ordinary concrete. When the number of freeze–thaw cycles is 50 times, the flexural strengths of MRC-10 and MRC-20 are 5.92 MPa and 5.54 MPa, respectively, 9.2% and 2.2% higher than PC, respectively. The total of 2.2% is due to the addition of rubber particles to increase the air content of the concrete mix, which provides space for the volume expansion when water freezes and weakens the stress generated by expansion.

The flexural strength of modified recycled rubber concrete after modification is higher than that of recycled rubber concrete because the rubber particles are modified to improve the hydrophobicity, make it easier to absorb water, and the cement particles around the rubber particles are more adequately hydrated, which improves the flexural strength of recycled rubber concrete. When the number of freeze–thaw cycles is 50 times, the flexural strength of MRSPC10-2, MRSPC10-4 and MRSPC10-6 are 6.04 MPa, 5.89 MPa and 5.03 MPa, respectively, in which the flexural strength of MRSPC10-2 is higher than that of MRC-10, which suggests that a small amount of straw admixture can improve the flexural strength of the recycled rubber concrete, because the straw powder can play the role of filling the pores and improve the concrete compactness, thus improving the compressive strength of concrete, while with the increase in straw admixture, the flexural strength of recycled rubber straw concrete gradually decreased, indicating that too much straw admixture hurts the flexural strength of recycled rubber straw concrete.

### 3.5. Microstructural Characteristics of Recycled Rubber Straw Concrete

**Figure 6:**
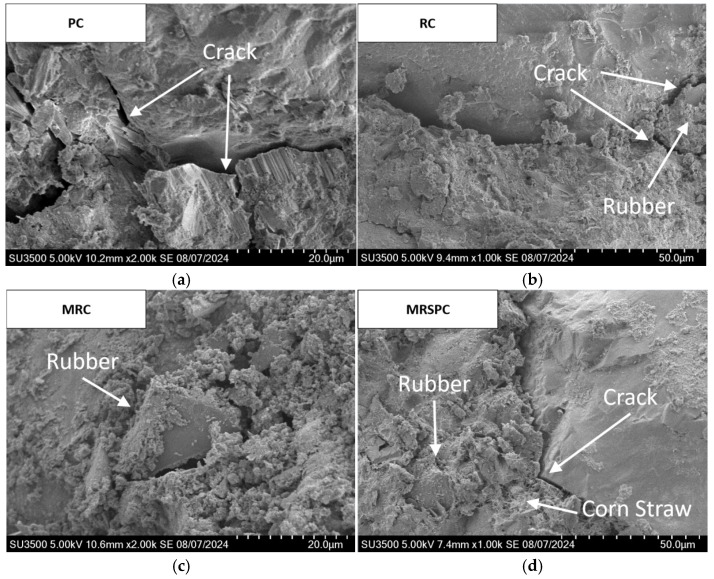
Micromorphology of recycled rubber straw concrete: (**a**) PC; (**b**) RC; (**c**) MRC; and (**d**) MRSPC.

SEM images of recycled rubber straw concrete are shown in Figure 6. Before SEM observations, the samples underwent an elaborate preparation process that included cutting, grinding, polishing, and necessary cleaning steps to ensure the accuracy and representativeness of the observations. Figure 6a shows many fine cracks and holes inside the ordinary concrete, which will become the weak point due to the stress concentration under external loading, and the cracks will further expand, leading to the destruction of the concrete. From Figure 6b, it can be seen that there are apparent boundaries in the interfacial transition zone between the rubber aggregate and cementitious materials of RC group, and the adhesion between rubber particles and cementitious materials is poor. Figure 6c shows that when the surface of rubber particles uses silane coupling agent for modification treatment, the interface hydration product is more intensive, and the hydration product closely attached to the surface of rubber particles, indicating that the use of silane coupling agent can effectively improve the interfacial transition zone adhesion strength, improve the compatibility of rubber particles and cement slurry, so that the thickness of the interfacial transition zone gradually becomes thinner, thus improving the mechanical properties of rubber aggregates cementitious materials. It can be seen from Figure 6d that after mixing straw powder, straw powder provides more attachment sites for hydration products while filling the pores, increases the area of generation of hydration products, and makes straw powder and cementitious tightly combined to hinders the development of cracks.

**Figure 7:**
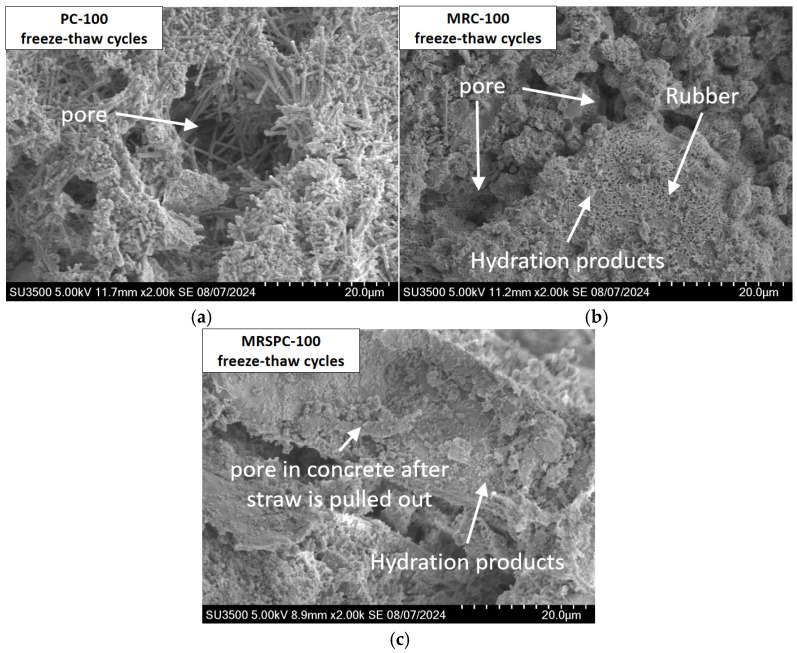
Micromorphology of recycled rubber straw concrete after 100 freeze–thaw cycles. (**a**) PC-100 freeze–thaw cycles; (**b**) MRC-100 freeze–thaw cycles; and (**c**) MRSPC-100 freeze–thaw cycles.

SEM was used to observe the microstructure of the recycled rubber straw concrete after the freezing and thawing process. The SEM image of PC after 100 freeze–thaw cycles is shown in Figure 7a. It shows that the internal pore structure of ordinary concrete becomes loose, and micro-cracks increase after 100 freeze–thaw cycles. Figure 7b shows that compared with ordinary concrete after freeze–thaw, rubber particles can introduce tiny bubbles, which form tiny pores in the concrete, which helps the water inside the concrete to migrate and release pressure during the freeze–thaw cycle, thereby reducing the internal damage caused by water freezing in the concrete. Moreover, the silane coupling agent-modified rubber concrete has a denser pore structure, better continuity and density of interfacial transition zone, and more C-S-H hydration products after the freeze–thaw cycle. These changes help improve the stability and mechanical properties of rubber concrete under the action of the freeze–thaw cycle. Figure 7c shows that some hydration products remain in the concrete after straw extraction, and compared with PC, there are fewer micro-cracks after a freeze–thaw cycle of MRSPC, which indicates that straw plays a specific role in filling and supporting the concrete, helping to reduce the pore size and increase the compactness of concrete.

In summary, the microstructure of concrete before the freeze–thaw cycle is usually relatively dense. After the freeze–thaw cycle, the microstructure of concrete deteriorates obviously. With the expansion of microcracks, the hydration products gradually changed from dense to loose. These changes cause the mechanical properties of concrete to decline. Because rubber particles can inhibit the deterioration of pores and straw powder can fill the pores, the density of reclaimed rubber straw concrete is better than that of ordinary concrete, and the cracks are smaller.

## 4. Neural Network Modeling

### 4.1. Introduction to the Model Database

In order to construct a prediction model for the flexural strength of recycled rubber straw concrete considering different mixing ratios, this paper selects straw admixture, rubber admixture quality, sand admixture, silane coupling agent admixture, and the number of freeze–thaw cycles, which have a more significant influence on the flexural strength of concrete, as input variables and flexural strength as output variable according to the test mixing ratios and test results. The total sample size for this training was 80 data sets. Among them, 24 data sets were randomly selected as test samples, accounting for 30% of the total sample size, and the remaining 56 data sets were used as training samples.

### 4.2. Model Evaluation Indicators

Different neural networks have different degrees of prediction for the same data. In order to better validate and compare the prediction ability between each model, the mean square error (RMSE) and the correlation coefficient (R) are used for the comparison. The computational equations are shown in Equations (1) and (2):

(1) $$RMSE=\sqrt{\frac{1}{n}\sum_{i=1}^{n}{\left(Y-Y^{*}\right)}^{2}}$$

(2) $$R=\frac{\sum_{i=1}^{n}\left(Y-\bar{Y}\right)\left(Y^{*}-{\bar{Y}}^{*}\right)}{\sqrt{{\sum_{i=1}^{n}\left(Y-\bar{Y}\right)}^{2}\sum_{i=1}^{n}\left(Y^{*}-{\bar{Y}}^{*}\right)}}$$

where:

n—number of samples for the test;
Y—test value;
$Y^{*}$—predicted value;
$\bar{Y}$—Test mean value;
${\bar{Y}}^{*}$—predicted mean value.

### 4.3. Test Model

The BP neural network uses a 3-layer model structure: input layer–implicit layer–output layer. The model structure is 5-L-1, based on the empirical Equation (3):

(3) $$L=\sqrt{m+n}+a$$

where:

L—number of nodes in the implicit layer;
m—number of nodes in the input layer;
n—number of nodes in the output layer;
a—constant, generally 1 to 10

**Figure 8:**
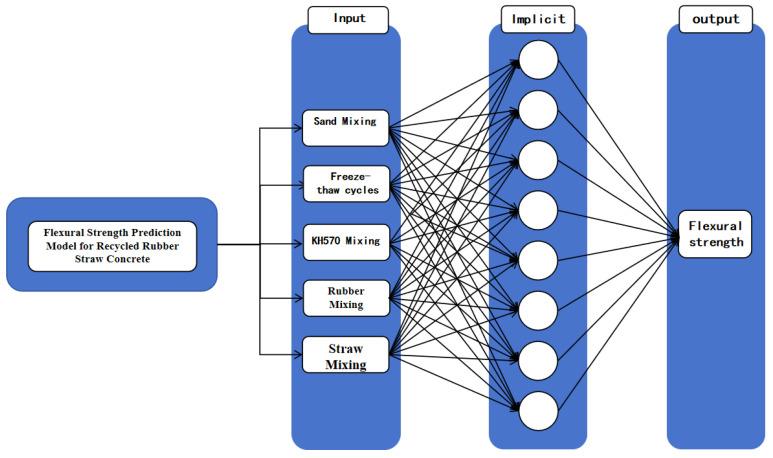
BP neural network structure.

Therefore, the value of the hidden layer is set between 4 and 13. The number of neuron nodes in the hidden layer of the neural network is selected to be eight according to repeated tests so that the final model structure is obtained as 5-8-1. The number of training times is set to be 1000, with a learning rate of 0.01, and the minimum error of the training target to be 0.000001. The structure of the BP neural network is shown in Figure 8.

PSO is an intelligent population search algorithm for nonlinear function optimization problems in multidimensional space, which generates a series of random particles during its initialization process. It takes this series of random particles as the solution object to make them move in the solution space to obtain the optimal solution in the solution process. The network parameters are optimized by updating the velocity and position of each particle according to its historical optimal position Pbest and global historical optimal position Gbest. Repeatedly perform the evaluation and update particles until the maximum number of iterations is reached or when the error (F1) of Gbest is lower than the pre-determined error Δ. PSO has notable features such as rapid convergence speed, high versatility, and outstanding global optimization ability. Using the PSO-BP optimization model can alleviate the problem of the BP neural network tending to local optimization and reduce the final prediction error. However, the performance of the PSO algorithm is more sensitive to the selection of inertia weight and acceleration coefficient, and PSO lacks a similar mutation mechanism, which may lead to insufficient population diversity. The control parameters in the PSO-BP algorithm affect the algorithm’s performance, and usually, choosing the appropriate control parameters is a complex optimization problem. There is no theoretical basis to guide the parameter selection. Generally speaking, the larger the population size, the more extensive the particle swarm search range will be, and it is easier to explore the global optimal solution, but the number that is too large will also increase the computation time. The general number is 20 to 50, and 50 is selected for this work. The acceleration constant is taken as c1 = c2 = 2.

**Figure 9:**
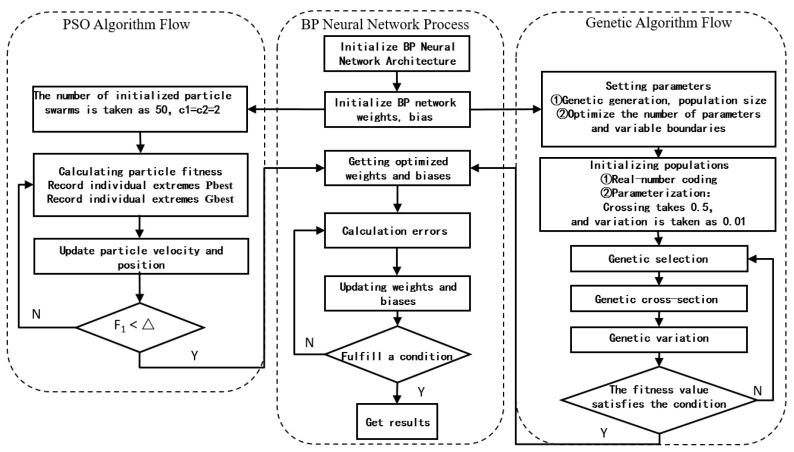
GA-BP and PSO-BP neural network design process.

The BP neural network using a gradient descent algorithm is prone to the disadvantage of falling into local minima, which affects the model’s prediction ability. The GA-BP neural network overcomes the disadvantage of the BP neural network, which is prone to falling into local minima, by introducing genetic algorithms to optimize the weights and thresholds of the BP neural network globally. This optimization process makes the GA-BP neural network more effective in finding the optimal solution and improves the network’s performance. A genetic algorithm is used to optimize the initial weights and thresholds of the neural network. In the genetic algorithm, the chromosomes in the population are constantly updated through selection, crossover, mutation, and other operations. Some individuals are eliminated according to the size of their fitness. Some individuals are selected to reproduce their offspring, and the algorithm is iterated several times until it converges to the best individual. GA-BP has good global search performance and can avoid falling into local optimal. GA optimization algorithm uses crossover and mutation operations to maintain population diversity and can comprehensively explore the search space. However, the convergence rate of GA is usually slower than that of PSO, especially when the sample size is large. The GA-BP neural network model is improved by a genetic algorithm in which the number of populations is set to 100. According to the repeated experiments, the model adopts 200 iterations, the crossover probability is generally between 0.25 and 1, the model selects 0.5, the mutation probability is generally between 0.01 and 0.1, and the experimental probability selects 0.01. The detailed algorithm flow of the PSO-BP and GA-BP models is shown in Figure 9.

### 4.4. Model Prediction Results

**Figure 10:**
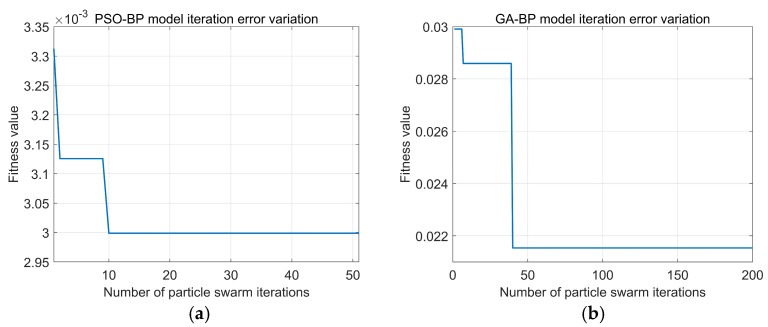
Convergence curves of different models: (**a**) PSO-BP; (**b**) GA-BP.

To verify the optimization model, a K-fold crossover algorithm was used to divide the dataset into 10 parts in this study. In the cross-validation process, one part of the dataset was set as the testing dataset, while the rest of the k-1 parts acted as the training dataset. In this study, k is set to 10, so 72 datasets are used as training sets for learning, and 8 datasets are used as test sets for testing. The convergence curves of PSO-BP and GA-BP are shown in Figure 10.

**Figure 11:**
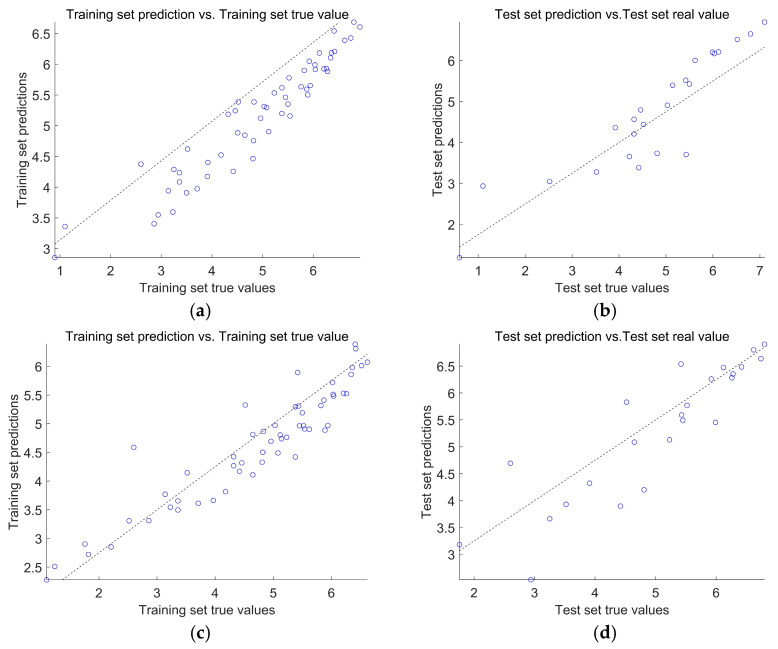

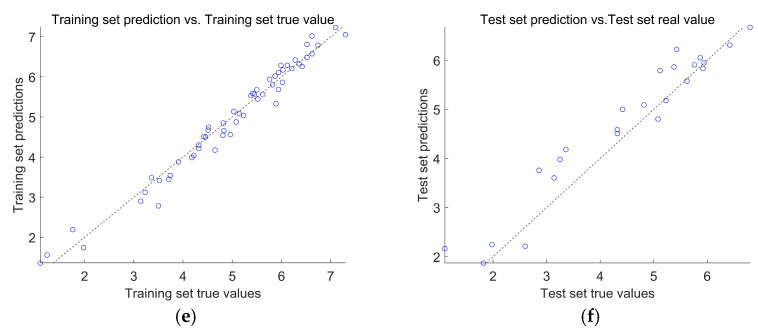
Schematic of the linear fit of the three neural network regressions: (**a**) BP Training set; (**b**) BP Test set; (**c**) GA-BP Training set; (**d**) GA-BP Test set; (**e**) PSO-BP Training set; and (**f**) PSO-BP Test set.

**Figure 12:**
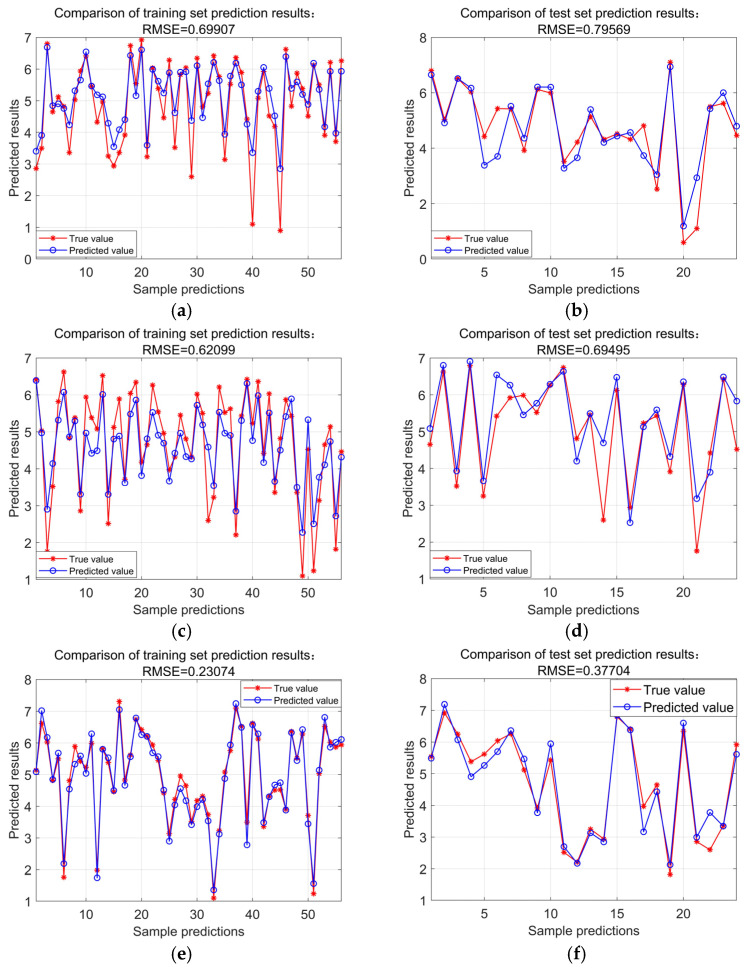
Prediction results of three neural network samples. (**a**) BP Training set; (**b**) BP Test set; (**c**) GA-BP Training set; (**d**) GA-BP Test set; (**e**) PSO-BP Training set; and (**f**) PSO-BP Test set.

Figure 10a shows that the PSO-BP model’s fitness value has been stable when iterated for the 9th time. Its fitness value has not changed in the subsequent iterations, and its optimal fitness value is 2.997 × 10^−3^ and has been in a convergent state. Figure 10b shows that the GA-BP model’s fitness value has been stable when iterated to the 40th time, its fitness value has not changed in the subsequent iterations, and its optimal fitness value is 2.153 × 10^−2^. Then, PSO-BP and GA-BP optimization models are used to predict the bending strength of concrete, and the results are compared with those predicted by the BP model. Then, PSO-BP and GA-BP optimization models are used to predict the bending strength of concrete, and the results are compared with those predicted by the BP model. The prediction status of the training and validation sets of the three models is shown in Table 2. It can be seen that the PSO-BP model has the smallest RMSE among the three models. Figure 11 shows the fitting effect of the three neural networks’ training and testing sets, in which it can be seen that the correlation between the testing values and the training values of the PSO-BP and GA-BP models is higher than that of the BP model. The correlation coefficient R is the largest, reaching 0.97 and 0.97 for the training and validation sets, while the GA-BP model is the second largest, and the BP model has the worst prediction performance. Figure 12 shows the sample prediction results of the three neural network models, from which it can be seen that the PSO algorithm and GA algorithm can optimize the BP neural network well under the use of the same BP network structural parameters. The PSO algorithm has higher computational efficiency in predicting the flexural strength of modified recycled rubber straw powder concrete compared to the GA algorithm. From the average error rate, it can be seen that the average error difference between the training set and test set of the BP neural network is significant, which indicates that the stability of model prediction is poor. The PSO-BP and GA-BP models have better stability with a minor difference between the average error rate of the training and test sets.

## 5. Case Study

The above study shows that the PSO-BP model has a better fitting effect. In order to provide a reference for engineering applications in cold regions, the frost resistance of recycled rubber straw concrete with different numbers of freeze–thaw cycles within the test range is further predicted. When a particular grade of concrete is needed for a project in a straw-rich area, straw samples are first taken locally. The rubber mixing amount, silane coupling agent mixing amount, sand mixing amount, and the target flexural strength of the concrete is inputted into the prediction model to obtain the mixing ratio and several freeze–thaw cycles of recycled rubber straw concrete with the required flexural strength. Example: when the project needs concrete with a flexural strength of 6.0 MPa, the PSO-BP neural network prediction model is completed by taking the flexural strength, rubber mixing amount, silane coupling agent mixing amount, sand mixing amount, and the number of freezing and thawing cycles as the input variables, and the straw mixing amount as the output variable. The prediction results are more accurate (training set R = 0.956, test set R = 0.962). Then, the determined rubber admixture, silane coupling agent admixture, sand admixture, the number of freeze–thaw cycles, and the flexural strength of the target concrete are used as the input variables, which are normalized with and sent to the established PSO-BP neural network prediction model. The inverse normalization is processed, and the predicted straw admixture can be outputted.

**Figure 13:**
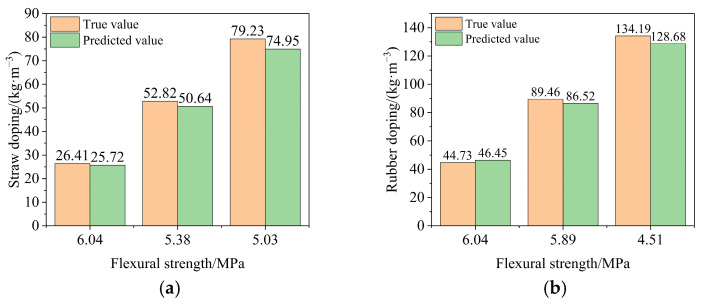
Predicted results of straw doping and rubber doping. (**a**) Comparison of straw doping projections and (**b**) comparison of rubber doping projections.

In this study, the model considers the flexural strength of 6.04 MPa, sand doping of 552 kg/m^3^, rubber doping of 44.8 kg/m^3^, 50 freeze–thaw cycles, and silane coupling agent doping of 0.891 kg/m^3^ as the input variables. The model obtains the output variable of straw doping of 25.72 kg/m^3^, and the flexural strengths of 5.38 MPa and 5.03 MPa, respectively, are taken as input variables. The corresponding predicted straw dosage is shown in Figure 13a. As the flexural strength increases, the required straw doping gradually decreases, and the prediction results are consistent with the actual situation. The error is within 7%, indicating that the prediction model with straw doping as the output variable has high accuracy. Similarly, a prediction model with rubber doping as the output variable can be established, and the predicted rubber doping is shown in Figure 13b.

**Figure 14:**
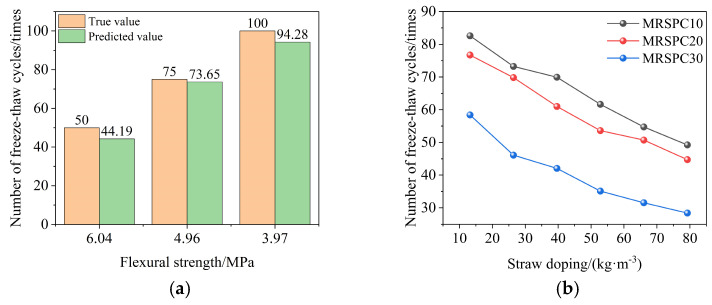
Predicted number of freeze–thaw cycles. (**a**) Comparison of different flexural strengths and (**b**) comparison of different straw and rubber doping.

Above all, a life prediction model with the number of freeze–thaw cycles as the output variable was established, fixing the straw doping at 26.41 kg/m^3^ to take straw doping, rubber doping, sand doping, silane coupling agent doping, and flexural strength as the input variables, and then the flexural strength of 6.04 MPa, 4.96 MPa, and 3.97 MPa was used as the input variables to obtain the corresponding predicted freeze–thaw cycles as shown in Figure 14a. The errors between the corresponding predicted results and the actual results were 11.62%, 1.80%, and 5.72%, with an average error of 6.38%, indicating that the prediction model can predict the number of freeze–thaw cycles of recycled rubber straw concrete with different flexural strengths more accurately. Subsequently, the corresponding predicted number of freeze–thaw cycles was obtained by taking straw and rubber admixtures as variables, respectively, as shown in Figure 14b. With the increase in straw and rubber doping, the number of freezing and thawing cycles to reach the corresponding flexural strength decreases, consistent with the test results. In conclusion, the straw mixing prediction model, rubber mixing prediction model, and life prediction proposed in this study based on engineering applications have good predictive ability and can predict the number of freeze–thaw cycles when the recycled rubber straw concrete reaches the corresponding flexural strength under different straw and rubber mixing, which can provide a reference value for the optimal design of mixing ratios and performance regulation of recycled rubber straw concrete.

## 6. Conclusions

A series of tests were conducted on recycled rubber straw concrete exposed to freeze–thaw cycles to analyze the effect of straw rubber coupling on the frost resistance of recycled rubber straw concrete. The study results provide guidance for applying recycled rubberized straw concrete in seasonal freezing zones. The main conclusions are as follows:

(1) When the number of freeze–thaw cycles is the same, the flexural strength of concrete decreases with the gradual increase in rubber mixing amount. When the number of freezing and thawing cycles is less, the flexural strength of ordinary concrete is higher than that of rubber concrete. When the number of freezing and thawing cycles is increased, the concrete with high rubber dosage can better slow the decline of the overall flexural strength, and the advantage of frost resistance becomes increasingly apparent. When the number of freezing and thawing cycles reaches 50 times, the flexural strength of concrete with 10% and 20% rubber dosage is 5.92 MPa and 5.54 MPa, respectively, 9.2% and 2.2% higher than that of ordinary concrete. The surface modification of rubber particles by silane coupling agent KH570 can effectively improve the hydrophobicity of rubber particles, increase the reactivity of the surface and cement paste, and improve the compactness of concrete.
(2) The relative dynamic modulus of elasticity of recycled rubber straw concrete with 2% straw doping was higher than that of recycled rubber concrete without straw doping, and it had good frost resistance. In comparison, 4% and 6% of straw doping hurt the frost resistance of recycled rubber straw concrete. Straw at 2% has a positive effect on the flexural strength of the concrete.
(3) The three neural network models established in this paper using sand doping, straw doping, rubber doping, silane coupling agent doping, and the number of freeze–thaw cycles as input variables have good predictive performance; the model correlation coefficients are all greater than 0.92, which can predict the flexural strength of recycled rubber straw concrete very well, and the predictive performance of the PSO-BP neural network model is better compared with that of the GA-BP neural network model.
(4) The PSO-BP model was utilized for the case study of engineering applications. The prediction of straw doping and rubber doping under the flexural strength of concrete with different engineering demands, as well as the prediction of the number of freeze–thaw cycles of concrete with different flexural strengths and different mixing ratios, were presented. The prediction results are close to the experimental results, indicating the model has good accuracy and stability.

## Figures and Tables

**Table 1:** Specimen proportioning parameters for each group.

Sample No.	Mix Proportion (kg/m^3^)								
	Water	Cement	Sand	Fine Aggregate	Coarse Aggregate	Rubber Particle	Straw Powder	Water Reducer	KH570
PC	195	487	613	340	794	0	0	0.49	0
RC-10	195	487	552	340	794	44.73	0	0.49	0
RC-20	195	487	491	340	794	89.46	0	0.49	0
RC-30	195	487	429	340	794	134.19	0	0.49	0
MRC-10	195	487	552	340	794	44.73	0	0.49	0.89
MRC-20	195	487	491	340	794	89.46	0	0.49	1.78
MRC-30	195	487	429	340	794	134.19	0	0.49	2.67
MRSPC10-2	195	487	552	340	794	44.73	26.41	0.49	0.89
MRSPC20-2	195	487	491	340	794	89.46	26.41	0.49	1.78
MRSPC30-2	195	487	429	340	794	134.19	26.41	0.49	2.67
MRSPC10-4	195	487	552	340	794	44.73	52.82	0.49	0.89
MRSPC20-4	195	487	491	340	794	89.46	52.82	0.49	1.78
MRSPC30-4	195	487	429	340	794	134.19	52.82	0.49	2.67
MRSPC10-6	195	487	552	340	794	44.73	79.23	0.49	0.89
MRSPC20-6	195	487	491	340	794	89.46	79.23	0.49	1.78
MRSPC30-6	195	487	429	340	794	134.19	79.23	0.49	2.67

**Table 2:** Parameters of evaluation metrics for the training and test sets of the three models.

Evaluation Indicators	BP		PSO-BP		GA-BP	
	Training Set	Test Set	Training Set	Test Set	Training Set	Test Set
Root mean square error (RMSE)	0.70	0.80	0.23	0.37	0.62	0.69
Correlation coefficient (R)	0.92	0.93	0.97	0.97	0.95	0.94
Average error rate	7.35	10.65	3.52	4.65	8.26	9.13

## Data Availability

The datasets used and/or analyzed during the current study are available from the corresponding author upon reasonable request due to privacy.
